# Underlying mechanisms of spatial distribution of prokaryotic community in surface seawater from Arctic Ocean to the Sea of Japan

**DOI:** 10.1128/spectrum.00517-25

**Published:** 2025-05-30

**Authors:** Ying Pan, Ye Tao, Xian Yang, Siyi Du, Hongguang Ding, Jiaxin Li, Hanwen Jia, Huaihai Chen

**Affiliations:** 1 State Key Laboratory of Biocontrol, School of Ecology, Shenzhen Campus of Sun Yat-sen University 582261 , Shenzhen, Guangdong, China; Yan'an University, Yan'an, Shaanxi, China

**Keywords:** microbial ecology, surface seawater, temperature

## Abstract

Microorganisms play critical roles in marine ecosystems, so understanding the factors shaping microbial communities across various oceanic regions is essential for predicting ecosystem resilience and biogeochemical cycles. This study investigated the marine prokaryotic communities across 22 stations spanning the Arctic Ocean, the Chukchi Sea, the Bering Sea, and the Sea of Japan, with an emphasis on how environmental factors shape these communities. Results showed that the microbial alpha diversity generally declines with increasing latitude, though Arctic Ocean stations exhibited higher Chao 1 indices compared to the Bering Sea. Beta diversity analyses revealed that temperature and salinity were key factors associated with community composition variation across latitudes. Proteobacteria and Cyanobacteria were the dominant phyla showing opposite distribution trends across sampling stations. Cold-adapted oligotrophs such as *Planktomarina* and the SAR11 clade thrived in Arctic waters, while *Sphingomonas,* known for pollutant degradation, was more abundant in the Sea of Japan. Temperature was positively correlated to the relative abundance of *Sphingomonas*. At broad spatial scales, stochastic processes dominated community assembly of microbial phylogenetic diversity, while in specific regions like the Arctic Ocean, deterministic homogeneous selection appeared to shape microbial communities; and temperature showed a pronounced influence on phylogenetic turnover across all samples. Co-occurrence networks identified several key taxa, such as *Polaribacter_1*, *Candidatus_Aquiluna*, and *NS5_marine_group*. Overall, the study underscores temperature’s role in shaping microbial community diversity, composition, and assembly processes across latitudinal gradients, highlighting unique community adaptations to extreme environments.

**IMPORTANCE:**

Microbes are the invisible engines of ocean health, recycling nutrients and sustaining marine life. This research helps us understand how climate factors like temperature shape these microscopic communities, which differ starkly between icy Arctic waters and warmer seas. As oceans warm due to climate change, microbial populations and their critical roles in cleaning pollutants or supporting food webs could shift dramatically. The study suggests Arctic microbes are uniquely adapted to cold, low-nutrient conditions, making them vulnerable to warming. By linking temperature to microbial diversity, this work provides clues to predict how marine ecosystems might respond to climate shifts, informing efforts to protect ocean biodiversity and processes vital to Earth’s carbon and nutrient cycles.

## INTRODUCTION

Microorganisms play fundamental roles in the biogeochemical cycling of marine ecosystems, influencing carbon (C), nitrogen, and other nutrient flows critical to ocean health and climate resilience (1). These microbes mineralize dissolved organic matter from phytoplankton in the euphotic zone, effectively channeling organic C to upper trophic levels and contributing significantly to the ocean’s biological carbon pump (2–4). Their rapid response to shifts in environmental conditions and global climate change also underscores their role as indicators of ecological health (5). Understanding the abundance and diversity of marine microorganisms across different regions is thus essential for predicting ecosystem resilience and biogeochemical stability under changing environmental conditions (2).

Recent large-scale ocean sampling efforts, including the Tara Oceans Expedition, have greatly advanced our understanding of marine microbial ecosystems across broad oceanic regions (6). The integration of advanced sequencing technology and oceanographic tools has enabled us to assess microbial diversity and functionality across vast marine landscapes (7, 8). However, vast areas of the ocean, particularly those with unique environmental conditions, remain underexplored. The Arctic Ocean, inhabited mainly by cold-adapted taxa and affected by complex dynamics, still has limited microbial data available to reveal the big picture of spatial distribution of prokaryotic community in the ocean, especially under current warming conditions that likely impact their community structure (8, 9). More importantly, the Bering Sea and the Bering Strait, the Arctic’s primary link to the Pacific, introduce nutrient-rich, lower-salinity waters to the Arctic’s upper layers, potentially affecting the regional microbial compositions (10). In contrast, the semi-enclosed Sea of Japan, influenced by surrounding human activity and warmer waters, spans from subpolar to subtropical zones, creating significant environmental variability in temperature and salinity. Thus, the microbial communities here may differ markedly from those in polar regions (11), and further investigation is needed to elucidate the underlying mechanisms. Research on Southern Ocean microbial responses to rapid warming and ice melt demonstrates that rising sea temperatures may reduce key taxa responsible for biogeochemical cycles while boosting heterotrophic populations, indicating lasting impacts on Antarctic marine microbial diversity by century’s end (12). Analysis of western Pacific microbial communities reveals that environmental conditions outweigh geographical distance in shaping microbial distribution patterns (13). Contrastingly, studies in the Subtropical Northwest Pacific show bacterial communities clustering by spatial proximity, suggesting location factors may outweigh environmental parameters in structuring these ecosystems (14). These findings highlight the necessity for expanded latitudinal sampling to clarify how environmental factors interact with geography in microbial community distribution. While landmark initiatives including the Tara Oceans expedition (sampling 210 global sites) and the International Census of Marine Microbes have generated unprecedented data sets through large-scale transoceanic sampling (6, 15), there is a notable scarcity of investigations spanning polar to subtropical marine latitudes, leading to theoretical barriers in understanding the biodiversity patterns across marine ecosystems. Furthermore, most of the previous studies mainly focused on the changes in microbial diversity and taxonomic composition. The assembly processes governing community organization remain markedly understudied.

To fill the above knowledge gaps, this study investigated prokaryotic communities across 22 surface seawater samples collected from the Arctic Ocean to the Sea of Japan. These sites cover a gradient distance of 4,000 km, including both remote and more accessible areas. Our objectives were to (i) characterize microbial community diversity and composition across diverse marine regions, (ii) explore mechanisms underlying prokaryotic assembly processes and interaction responding to large spatial variation, and (iii) elucidate the underlying environmental determinants that significantly correlate to the spatial distribution of prokaryotic communities.

## MATERIALS AND METHODS

### Study area and sample collection

**Fig 1:**
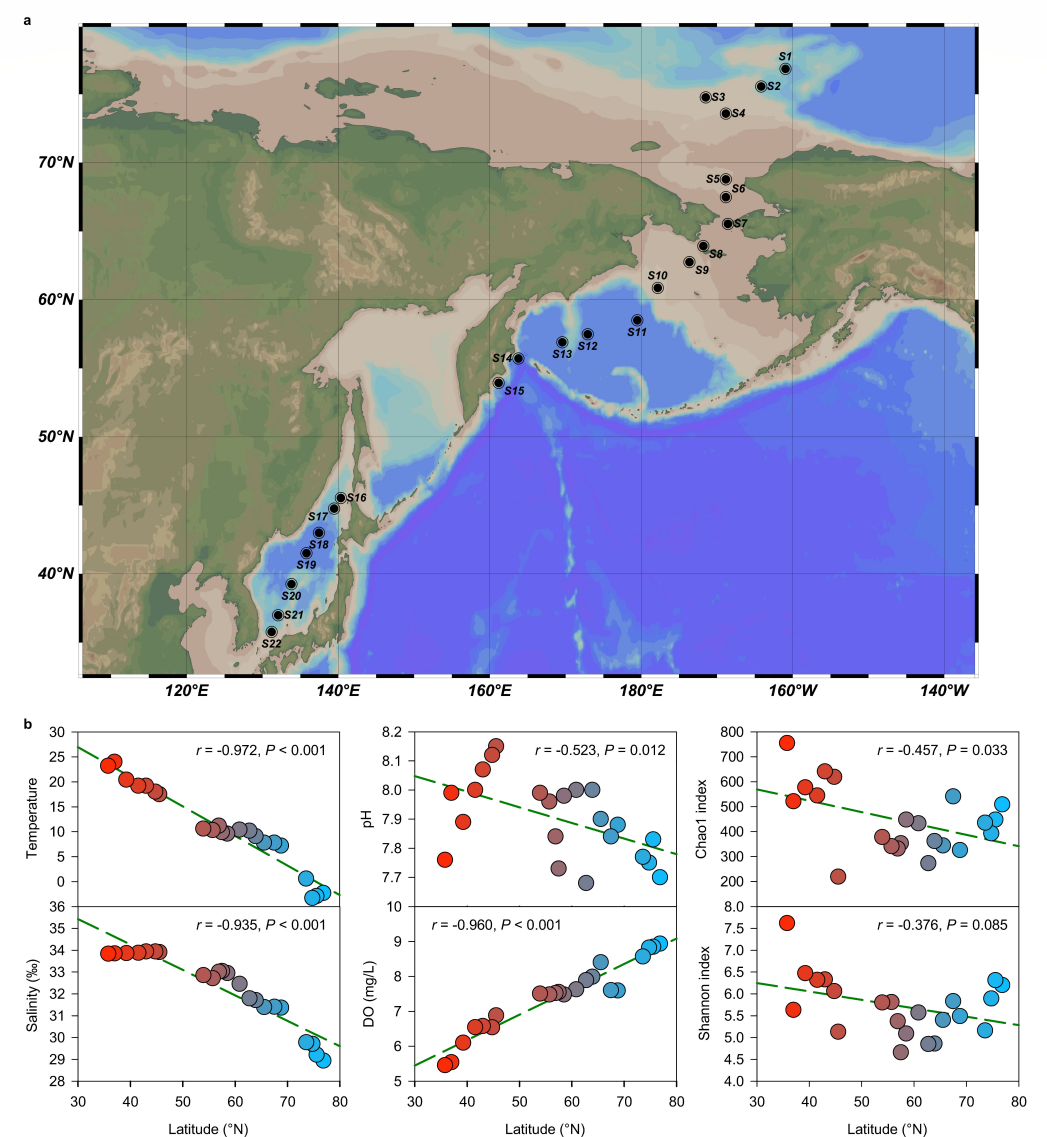
(**a**) 22 sampling stations of the Arctic Ocean, Bering Strait, Bering Sea, and Sea of Japan in August 2018; (**b**) Pearson’s correlations of latitudes versus temperature, pH, alpha diversity of Chao1 index in surface seawater collected from different sampling stations. Pearson’s correlation coefficient (*R*) and statistical significance (*P*) are displayed.

This interdisciplinary survey was conducted via China’s 9th Arctic research expedition aboard the icebreaker Xuelong in the summer of 2018, spanning 22 stations from the east margin of the Canadian Basin (Arctic Ocean) through the Chukchi Sea to the Bering Sea and the Sea of Japan (35–76°N, 160°W–130°E) (Fig. 1a). Surface seawater samples were collected at each station (Table 1).

**TABLE 1:** Environmental characteristics, alpha diversity indices of prokaryotic communities in surface marine water from different sampling stations

Station	Longitude	Latitude	Temp (°C)	Salinity (‰)	DO^*a*^ (mg/L)	pH	TOC^*b*^ (mg/L)	NH_4_^+^ (mg/L)	NO_3_^–^ (mg/L)	Chao1	Shannon
S1	160°58′W	76°51′N	−2.1	28.94	8.94	7.70	4.22	0.231	0.113	509	6.20
S2	164°10′W	75°34′N	−2.2	29.21	8.85	7.83	5.23	0.227	0.111	447	6.31
S3	171°29′W	74°46′N	−2.3	29.71	8.82	7.75	9.27	0.100	0.063	393	5.89
S4	168°50′W	73°35′N	0.6	29.78	8.57	7.77	5.23	0.112	0.068	435	5.16
S5	168°50′W	68°47′N	7.2	31.36	7.60	7.88	1.69	0.196	0.100	326	5.49
S6	168°50′W	67°29′N	7.8	31.40	7.60	7.84	15.85	0.173	0.091	541	5.83
S7	168°33′W	65°32′N	7.8	31.39	8.40	7.90	17.87	0.250	0.120	344	5.40
S8	171°48′W	63°55′N	9.1	31.71	7.99	8.00	1.69	0.138	0.078	362	4.86
S9	173°38′W	62°45′N	10.2	31.79	7.89	7.68	7.25	0.330	0.151	273	4.85
S10	177°49′W	60°52′N	10.4	32.45	7.63	8.00	9.27	0.269	0.127	433	5.57
S11	179°29′E	58°30′N	9.6	32.95	7.48	7.98	17.37	0.066	0.050	447	5.09
S12	172°56′E	57°29′N	9.9	33.04	7.55	7.73	7.76	0.123	0.072	353	4.66
S13	169°35′E	56°54′N	11.2	33.01	7.52	7.84	1.69	0.204	0.103	331	5.37
S14	163°47′E	55°43′N	10.3	32.71	7.48	7.96	2.70	0.173	0.091	341	5.81
S15	161°10′E	53°36′N	10.6	32.85	7.51	7.99	9.27	0.200	0.101	378	5.80
S16	140°21′E	45°32′N	17.5	33.91	6.88	8.15	11.80	0.219	0.108	219	5.13
S17	139°27′E	44°46′N	18.0	33.93	6.54	8.12	8.26	0.200	0.101	619	6.06
S18	137°27′E	43°00′N	19.2	33.94	6.58	8.07	1.69	0.165	0.088	641	6.33
S19	135°49′E	41°31′N	19.2	33.87	6.54	8.00	4.22	0.085	0.057	544	6.32
S20	133°50′E	39°15′N	20.4	33.86	6.10	7.89	16.86	0.115	0.069	577	6.47
S21	132°05′E	36°59′N	24.0	33.85	5.54	7.99	5.73	0.204	0.103	521	5.63
S22	131°13′E	35°46′N	23.2	33.83	5.46	7.76	8.26	0.096	0.062	755	7.62

^*a*^ DO, dissolved oxygen.

^*b*^ TOC, total organic carbon.

Approximately 2 L of surface seawater was collected and filtered at each site (11). Temperature, salinity, pH, and dissolved oxygen (DO) were measured on board using a thermometer, a refractometer (Atago S-10, Japan), a pH meter (E.W. System Soil Tester, Japan), and a DO meter (Orion Star A223, Thermo Scientific, USA), respectively. Subsamples (~0.5 L) were stored at 4°C for later analysis of total organic carbon (TOC), ammonium (NH_4_^+^), and nitrate (NO_3_^−^). TOC was measured using a spectrophotometer (model UV-1206, Schimadzu, Japan) after oxidation by potassium dichromate (K_2_Cr_2_O_7_) and potassium permanganate (KMnO_4_). NH_4_^+^ and NO_3_^−^ were determined by the Flow Injection Analyzer (Lachat QuikChem Method 8000, USA). Additional seawater (~1.5 L) was filtered through 0.22 µm polycarbonate filters, and the filters with microorganisms were stored at −80°C prior to DNA extraction and prokaryotic community analysis.

### DNA extraction, amplification, and sequencing processing

The extractions of DNA were performed using the FastDNA spin kit for soil (MP Biomedical, USA) according to the manufacturer’s instructions. DNA integrity was verified on a 1.2% agarose gel with the DNA purity and concentration determined using an ND-2000C spectrophotometer (NanoDrop, USA). The V4 hypervariable region of the 16S rRNA gene was amplified using the 515F/806R primer pair, with a specific 6 bp barcode added to the reverse primer for each sample. The amplification reaction mixture (25 µL total volume) included 12.5 µL of 2× Phusion High-Fidelity PCR Master Mix (New England Biolabs), 0.2 µM of each primer, and 10 ng of template DNA. The reaction was performed on a T100 Thermal Cycler (Bio-Rad, USA) with an initial denaturation at 98°C for 1 min, followed by 30 cycles of denaturation at 98°C for 10 s, annealing at 50°C for 30 s, elongation at 72°C for 30 s, and a final extension step at 72°C for 5 min. Sequencing was performed using the Illumina MiSeq platform with the TruSeq Nano DNA LT Library Prep Kit (Illumina, USA).

The raw sequencing reads were demultiplexed according to their specific barcodes and imported into Quantitative Insights into Microbial Ecology version 2 (QIIME 2, version 2019.4) for further processing. The plugin DADA2 within QIIME 2 was used for quality control through the following sequential steps: (i) filtering of low-quality reads (Q-score <20), (ii) paired-end read merging with a minimum overlap of 12 base pairs, (iii) removal of chimeric sequences, and (iv) removal of singleton amplicon sequence variants (ASVs) (i.e., ASVs with a total read count of 1 across all samples). This workflow generated ASVs clustered at 100% sequence similarity. Taxonomic classification of ASV representative sequences was performed using a pre-trained Naive Bayes classifier in QIIME 2, referencing the Silva 16S rRNA database (Release 132) (16). The phylogenetic tree was constructed using the approximately maximum likelihood algorithm implemented in FastTree. To enable the uniformity of sequencing depth across samples in downstream analyses, rarefaction was performed by randomly subsampling each sample to 22,428 reads (95% of the minimum sequencing depth) prior to alpha/beta diversity calculations. This normalization approach ensures comparability of prokaryotic community metrics across samples.

### Microbial community assembly processes and co-occurrence networks

The nearest-taxon index (NTI) and beta nearest-taxon index (βNTI) were used to quantify the degree of phylogenetic clustering/dispersion degree within a single community and the phylogenetic distance between pairwise communities, respectively. The NTI values were calculated as the differences between the observed phylogenetic distances of the observed communities and the mean phylogenetic distances of the null communities, divided by the standard deviation of the null distribution (17). The NTI > 2 or NTI < −2 indicates that the community is more phylogenetically distantly/closely related than expected by chance, while the NTI values between −2 and + 2 indicate the community assembly is governed by dispersal-related or stochastic processes (18). The βNTI values were calculated by measuring the number of standard deviations between observed beta mean nearest-taxon distance (βMNTD) and the mean of the null model (19, 20). βNTI values exceeding +2 or below −2 indicate significant phylogenetic turnover than expectations, implying a strong influence of deterministic processes, specifically heterogeneous selection (exceeding +2) or homogeneous selection (below −2). βNTI values between −2 and +2 suggest that observed phylogenetic composition differences arise from stochastic processes, such as dispersal limitation, homogenizing dispersal, and other undominated processes (20).

To uncover the potential interactions among taxa within prokaryotic microbial communities, the network based on pairwise Spearman’s rank correlations was constructed at the genus level using the Molecular Ecological Network Analysis Pipeline (http://ieg4.rccc.ou.edu/MENA/) and Cytoscape (version 3.91). We summarized several parameters, including average node connectivity (avgK), average clustering coefficient (avgCC), geodesic distance (GD), geodesic efficiency (E), harmonic geodesic distance (HD), and modularity, to describe the network’s topology. Specifically, avgK, avgCC, GD, E, and HD serve as indicators of network efficiency. The modularity index provides insight into whether the network exhibits a modular structure.

### Statistical analysis

The principal component analysis (PCA) was conducted to assess the variation and correlation of the environmental variables (temperature, salinity, DO, NO_3_^-^, NH_4_^+^, TOC, pH) among different sampling stations. For alpha diversity of the microbial community, Chao1 and Shannon indices were calculated before sequencing rarefaction to 22,428 based on 95% of the minimum sequence number obtained from all samples. For beta diversity, distance-based redundancy analysis (dbRDA), principal coordinates analysis (PCoA), and Mantel’s test were conducted to investigate the variation of microbial community structure based on the Bray-Curtis distances calculated across all samples as well as their overall correlations with environmental variables (temperature, salinity, DO, NO_3_^-^, NH_4_^+^, TOC, pH). Additionally, dbRDA, PCoA, and Pearson’s correlations were also used to assess the variations of phylogenetic turnover, as represented by βMNTD values.

## RESULTS

### Environmental characteristics

**Fig 2:**
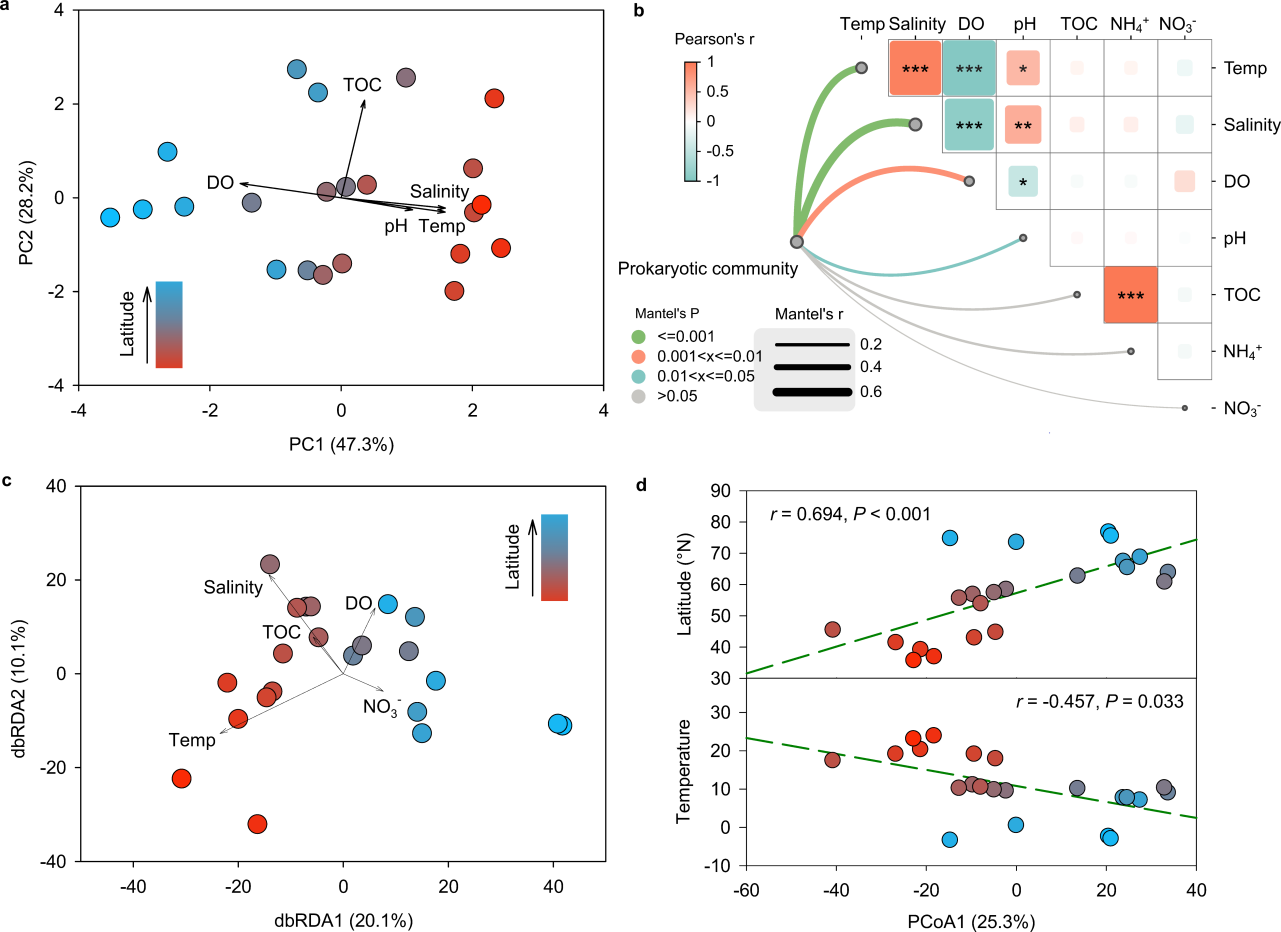
(**a**) PCA of environmental characteristics in surface marine water from different sampling stations; (**b**) Mantel’s test of environmental characteristics with prokaryotic community composition in surface marine water from different sampling stations; (**c**) dbRDA of prokaryotic community composition based on the Bray-Curtis similarity matrix in surface marine water from different sampling stations; (**d**) Pearson’s correlation of PCoA1 with temperature and latitude in surface seawater collected from different sampling stations. Vectors represent the explanatory variables of environmental characteristics. Pearson’s correlation coefficient (*R*) and statistical significance (*P*) are displayed.

Surface seawater temperature ranged from –2.3°C in the Canadian Basin (Arctic Ocean) to 24.0°C in the Sea of Japan, showing a significant negative correlation with the latitude across all sampling stations (*r* = −0.97, *P* < 0.001; Table 1, Fig. 1b). Similarly, pH values showed a weaker but still significant negative correlation (*r* = −0.52, *P* = 0.012), with the highest value (pH = 8.15) in the Sea of Japan (S16) and lowest (pH = 7.68) near the Bering Sea (S9). Salinity also showed a significant negative correlation (*r* = −0.93, *P* < 0.001) with the latitude, whereas the DO showed a significant positive correlation (*r* = 0.96, *P* < 0.001). Concentrations of TOC (1.69–17.87 mg/L), NH_4_^+^ (0.066–0.330 mg/L), and NO_3_^–^ (0.050–0.151 mg/L) varied among different sampling sites but did not show a consistent trend against the latitude. PCA and Pearson’s correlations showed that environmental characteristics generally separated among sampling stations (Fig. 2a), with TOC and NH_4_^+^ positively associated with each other. The temperature and salinity exhibited a positive correlation with each other, while both parameters showed positive correlations with pH values but negative correlations with dissolved oxygen concentrations (Fig. 2b).

### Microbiome community diversity and composition

A total of 682,470 high-quality 16S rRNA reads (23,609 to 37,471 reads per sample) were clustered into 5,212 ASVs at 100% sequence similarity. Based on the 22,428 sequences randomly selected from each sample, the alpha diversity index of Chao1 showed a significant decreasing trend with increasing latitudes, with the highest value (755) observed in S22 of the southern Sea of Japan (Fig. 1b). Mantel’s test results showed that temperature and salinity were the primary parameters influencing prokaryotic community structure (Fig. 2b). The dbRDA based on Bray-Curtis similarity revealed an obvious spatial pattern along the sampling latitudes, which were most significantly associated with temperature and salinity (Fig. 2c). In particular, PCoA1 (25.3%) was positively related to latitude (*r* = 0.69, *P* < 0.001) but negatively associated with temperature (*r* = –0.46, *P* = 0.033) (Fig. 2d).

**Fig 3:**
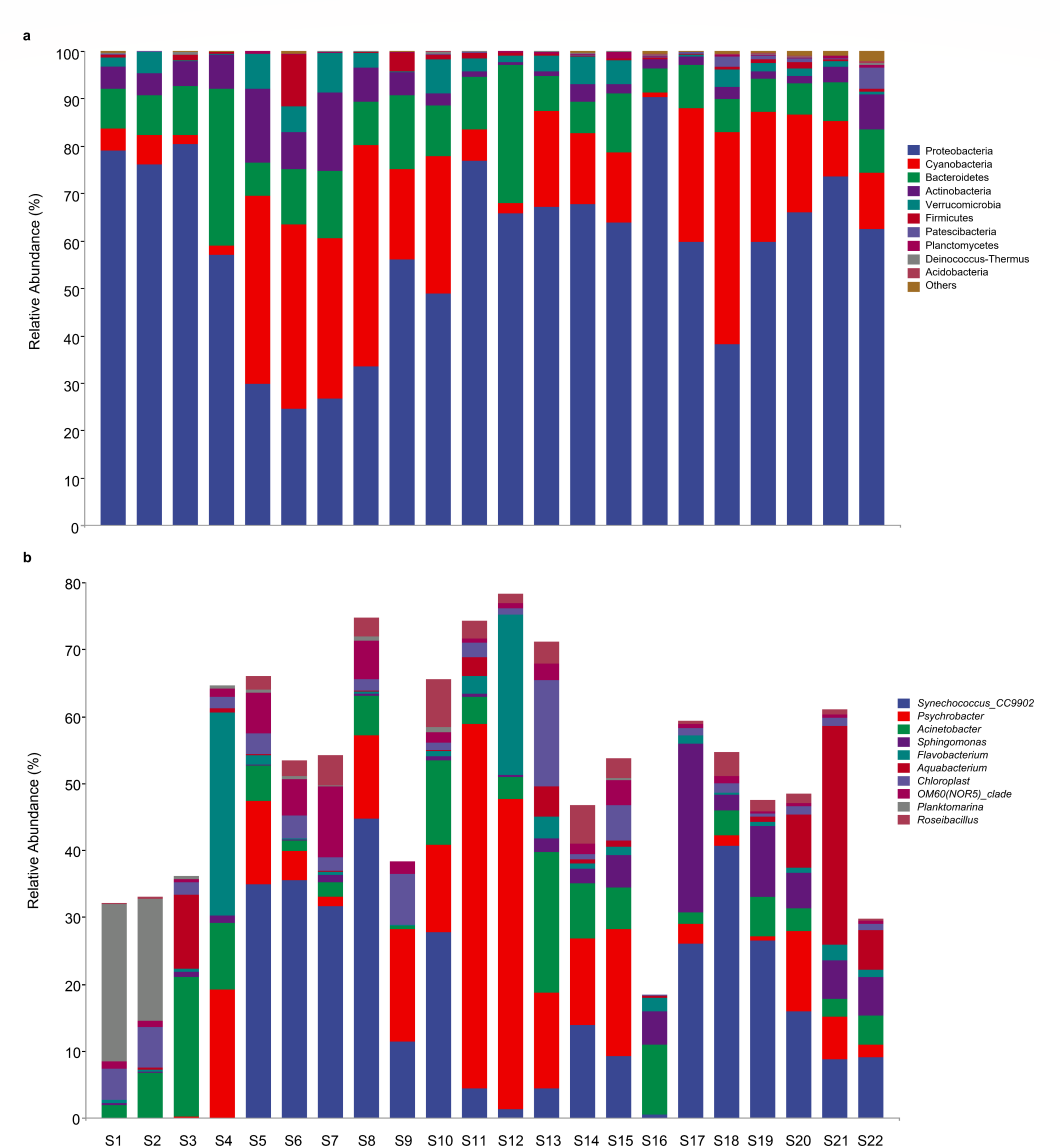
Relative abundances of major taxa at the phylum levels (**a**) and top 10 genera (**b**) of prokaryotic community in surface marine water from different sampling stations.

**Fig 4:**
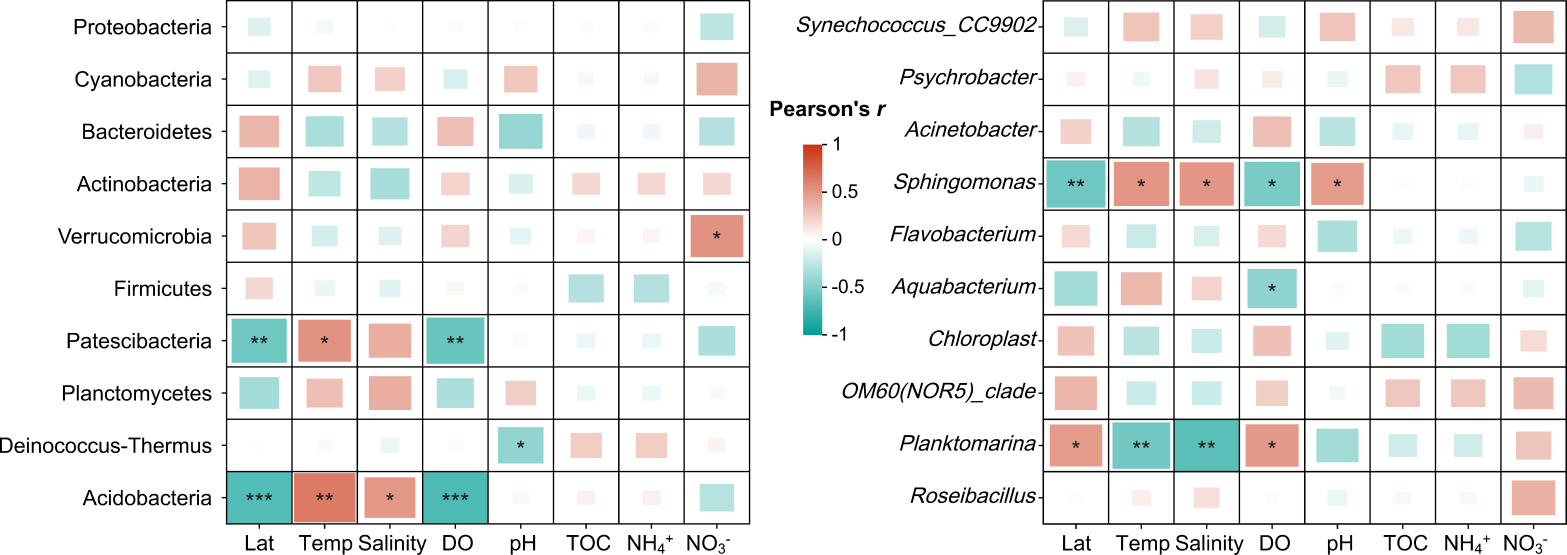
Pearson’s correlation of relative abundances of major taxa at the phylum and genus levels of prokaryotic community with environmental characteristics in surface seawater collected from different sampling stations. Pearson’s correlation coefficient (*R*) and statistical significance are indicated in the graph (*, *P* < 0.05; **, *P* < 0.01; ***, *P* < 0.001).

For prokaryotic community composition, a total of 32 phyla (including candidate divisions) were identified (Fig. 3a). Specifically, Proteobacteria, mainly characterized by Alphaproteobacteria (26%) and Gammaproteobacteria (33%), was the most abundant phylum (accounting for an average of 59%) across all samples. However, its relative abundance declined to 37% near the Bering Strait (S5–S10), while Cyanobacteria, the second most dominant phylum (19% on average), increased to 33% (Fig. 3a). Notably, the relative abundances of Patescibacteria and Acidobacteria were positively correlated with temperature and negatively with latitudes and DO (Fig. 4). At the genus level, *Synechococcus-CC9902* (belonging to Cyanobacteria), the most abundant taxa, was significantly lower in S1–S4 near the Arctic Ocean but significantly higher in S5–S10 (averagely 31%) in the Bering Strait (Fig. 3b). Meanwhile, *Psychrobacter* (Gammaproteobacteria) thrived in the Bering Strait/Bering Sea (S5–S15), and *Sphingomonas* (Alphaproteobacteria) showed higher abundances in the Sea of Japan (S16–S22). Moreover, *Sphingomonas* showed positive associations with temperature, salinity, and pH values, while *Planktomarina* exhibited negative correlations with temperature and salinity (Fig. 4).

### Microbial community assembly mechanisms and co-occurrence networks

**Fig 5:**
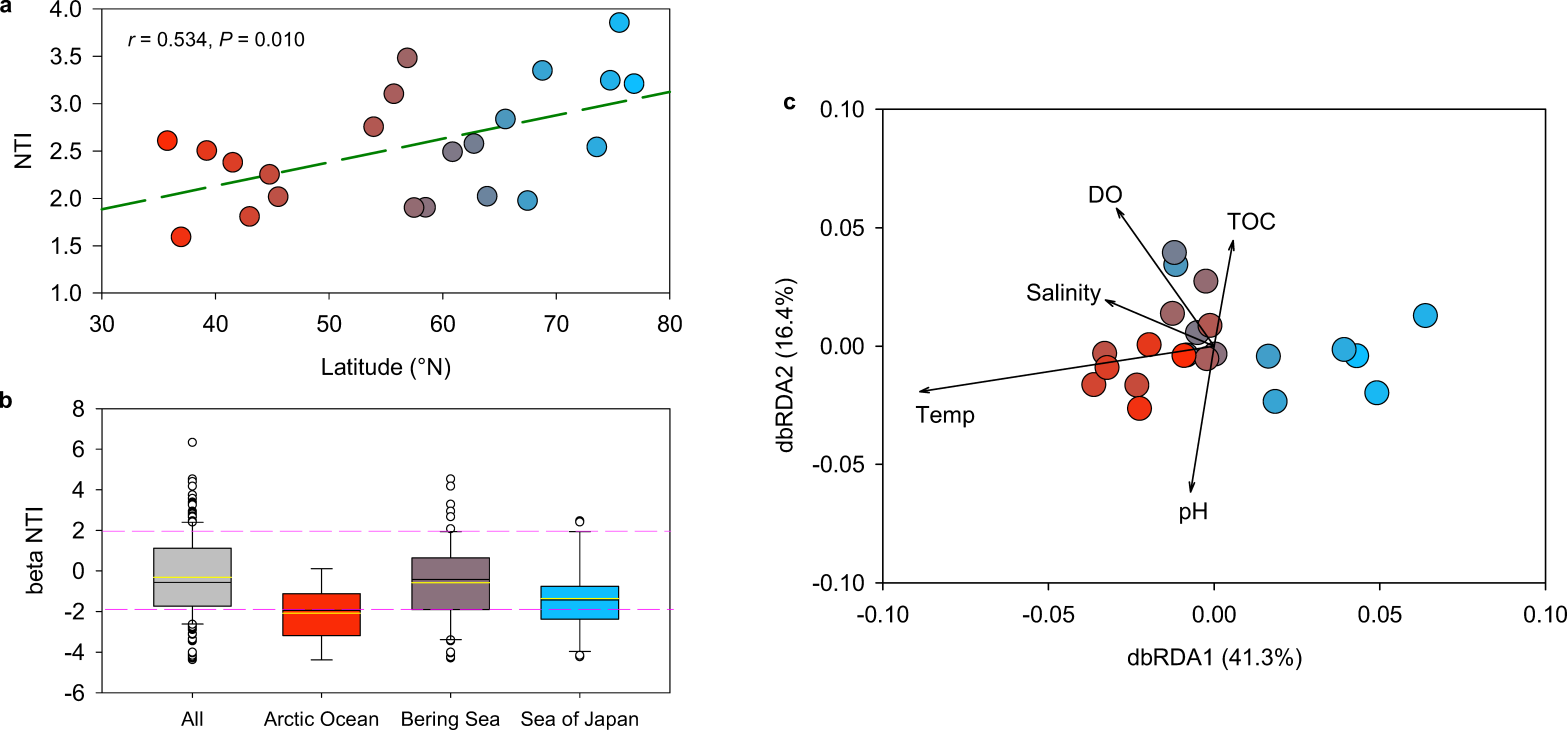
(**a**) Pearson’s correlation of weighted NTI with latitudes of different sampling stations; (**b**) Boxplots of the βNTI between communities in surface seawater collected from different sampling stations; (**c**) dbRDA of βMNTD between communities in surface seawater collected from different sampling stations. Vectors represent the explanatory variables of environmental characteristics. Pearson’s correlation coefficient (*R*) and statistical significance (*P*) are displayed.

The NTI values for all samples exceeded 1.5, with most surpassing 2 (Fig. 5a), indicating that the prokaryotic communities in these samples are more phylogenetically clustered than expected by chance, which infers most of the communities were deterministically structured (e.g., by environmental filtering). The NTI values also showed a slightly increasing trend with the latitude (*r* = 0.53, *P* = 0.01; Fig. 5a), suggesting a stronger environmental filtering effect in the polar regions.

The βNTI values between pairwise communities were also calculated to infer the predominant processes driving community assembly. Results showed that over 70% of the βNTI values were between −2 and +2 (Fig. 5b), suggesting that stochastic processes (e.g., ecological drift and random dispersal) largely influence community assembly in these marine environments. Meanwhile, lower βNTI values (average value < −2) were observed in pairwise samples collected in the Arctic Ocean than in the Bering Strait and Sea of Japan, suggesting that homogenous selection plays a more significant role in shaping community structure in extreme habitats. The dbRDA based on pairwise βMNTD also revealed a remarkable spatial distribution of samples along the sampling latitudes, which were most significantly associated with temperature in dbRDA1 (41.3%) (Fig. 5c), suggesting a key role of temperature as the most influential factor in shaping microbial phylogenetic diversity.

**Fig 6:**
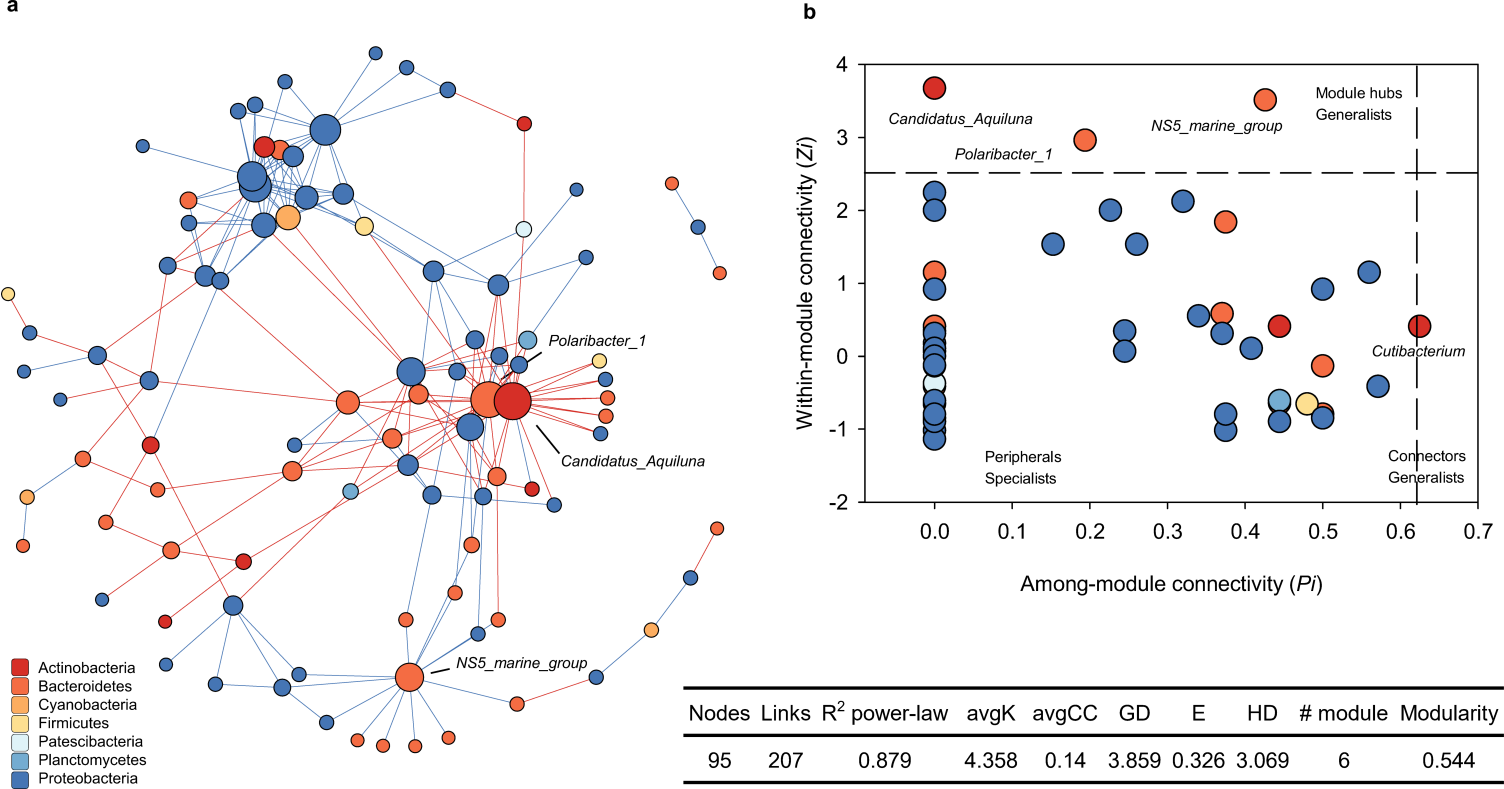
(**a**) Co-occurrence networks of prokaryotic community with basic network properties based on taxa at the genus levels; (**b**) node distribution of module-based topological roles determined by the scatter plot of within-module connectivity (*zi*) and among-module connectivity (*Pi*) of prokaryotic taxa at the genus levels. The threshold values of *zi* and *Pi* for categorizing were 2.5 and 0.62, respectively.

Co-occurrence network analysis identified potential interactions among taxa, with 95 nodes and 207 links (ρ > 0.8). Three key taxa, including *Polaribacter_1* (Bacteroidetes), *Candidatus_Aquiluna* (Actinobacteria), and *NS5_marine_group* (Bacteroidetes), were identified to be the module hubs generalists (Fig. 6b). The other detailed topological parameters, including *R*^2^ of power-law, average degree (avgK), avgCC, average path distance (GD), geodesic efficiency (E), harmonic geodesic distance (HD), and number of modules and modularity index, were also summarized (Fig. 6b).

## DISCUSSION

### Trends in marine prokaryotic diversity

This study investigated prokaryotic diversity patterns across latitudinal gradients, revealing a significant negative correlation between latitude and alpha diversity as measured by the Chao1 index (*r* = –0.457, *P* = 0.03). This trend aligns with previous findings in the Arctic Ocean and Bering Sea (8) and south of the Bering Strait (21, 22). The observed latitudinal diversity gradients support the metabolic theory of ecology, linking microbial diversity to kinetics of metabolism from the individual to the ecosystem scales (21, 23).

However, deviations from this pattern were observed in the Arctic Ocean stations, where Chao1 indices were notably higher than in the Bering Sea, suggesting incomplete explanations of the metabolic theory in microbial diversity patterns across large scales (24). The physiological tolerance hypothesis offers a complementary perspective, proposing that microbial species are more adapted to extreme environmental conditions, such as polar or tropical regions, rather than the intermediate environments with moderate selective pressures (8, 25). Supporting this, previous research identified 9.5°C as a critical temperature threshold dividing cold- and warm-adapted microbial communities (26). The distinct community structure observed in Arctic Ocean stations (S1–S4) compared to low-latitude sites (S16–S22) further reflected the role of cold temperature as a strong environmental filter (8). Furthermore, a recent biogeochemical study by Nishino et al. (10) based on data from the Synoptic Arctic Survey revealed anomalous hydrographic conditions in the western Arctic Ocean caused by the intrusion of Atlantic-origin water masses into the Pacific Arctic sector, which also provides a plausible mechanistic explanation for the “anomalous Arctic diversity” patterns.

In the mid-latitude regions (S5–S10), dynamic environmental factors, such as light availability and nutrient fluxes, contribute to seasonal microbial turnover. For instance, during phytoplankton blooms, bloom-associated plankton such as cyanobacteria can dominate the community by outcompeting other microorganisms, thereby reducing overall diversity. The prevalence of cyanobacteria observed in the Bering Strait (Fig. 3) aligns with this phenomenon, as blooms during the sampling period likely crowded out other microbial taxa, creating localized biodiversity minima (27). These results highlight the interplay between environmental gradients, seasonal dynamics, and microbial adaptation in shaping prokaryotic diversity across marine ecosystems.

### Characteristics of microbial communities in different regions

The Arctic Ocean represents a uniquely extreme environment on Earth, characterized by persistently low temperatures, limited carbon availability, and highly seasonal light conditions, collectively leading to the distinctive evolutionary adaptations of the microbial communities in the Arctic surface waters (28). Among these specialized communities, the genus *Planktomarina* (order Rhodobacterales) represents a key indicator of surface waters under sea ice cover (8). This genus was notably enriched at the northern Chukchi Sea sampling stations (S1 and S2), where seasonal sea-ice melt might influence community composition. In addition to *Planktomarina*, the SAR11 clade, affiliated with Alphaproteobacteria and known for its adaptation to oligotrophic (nutrient-poor) systems, was more abundant in the Arctic Ocean than in other regions. In fact, SAR11’s success in these nutrient-poor and cold conditions can be attributed to its efficient utilization of small organic molecules (29). At stations S1 and S2, where surface waters were cold and nutrient-limited due to freshwater inputs from ice melt driven by the Beaufort Gyre, SAR11 exhibited significantly higher relative abundance. Conversely, the southern Chukchi Sea (stations S3 and S4) displayed enrichment of Bacteroidetes, a group sensitive to cold water and phytoplankton blooms but adapted to nutrient-rich environments (30, 31). The inflow of nutrient-dense Pacific waters into the southern Chukchi Sea shelf region brings both warmth and inorganic nutrients, promoting the growth of Bacteroidetes and supporting a more nutrient-dependent community structure (32).

Bering Strait serves as the sole Pacific gateway to the Arctic Ocean, with water masses flowing predominantly northward, driven by a pressure gradient between the North Pacific and Arctic Oceans (33). In the northern Bering Strait (S5–S10), the phylum Cyanobacteria and its affiliating genus *Synechococcus-CC9902* became obviously predominant, and their average proportions in these stations reached 40%. As a group of autotrophic bacteria, Cyanobacteria is renowned for their carbon fixation via oxygenic photosynthesis (34). Certain cyanobacteria species also exhibited biological nitrogen (N) fixation capability, such as the unusual N_2_-fixing unicellular cyanobacteria (UCYN-A)/haptophyte symbiosis and *Synechococcus* spp., which played critical roles in the nitrogen cycle of marine ecosystems (35, 36). The significant presence of Cyanobacteria and *Synechococcus-CC9902* (reaching 40%) suggests elevated nitrogen fixation efficiency in this region, warranting further investigation. In contrast, the southern Bering Sea (S11–S15) exhibited distinctly different prokaryotic community compositions, likely due to hydrographical and biological variations between the northern and southern regions, demarcated around 60°N (37). In particular, the genus *Psychrobacter* (belonging to Gammaproteobacteria) flourished greatly, with a relative abundance exceeding 50% at station S11. *Psychrobacter* species, which are adapted to a wide range of temperatures and salinities (38), typically utilize simple organic substrates, such as organic acids and amino acids, rather than complex polysaccharides (39). The notably high TOC concentration at station S11 (17.37 C mg/L) likely provided a favorable environment for the proliferation of *Psychrobacter*. Additionally, the metabolism of seabirds and fish in this productive region, which hosts approximately 80% of the seabird population in the USA, may supply uric acid as a nitrogen and energy source, further supporting the growth of *Psychrobacter* (39). This correlation between seabird activity and microbial composition in the southern Bering Sea merits further investigation.

The Sea of Japan, a marginal sea of the western Pacific Ocean, serves as a crucial geopolitical and economic hub, connecting the countries of East Asian nations via major ports like Vladivostok, Nakhodka, and Pusan (40). However, its proximity to these industrial and shipping activities renders it vulnerable to pollution, including catastrophic oil spill incidents, which release thousands of tons of hydrocarbons into the marine environment (41). In our survey, the genus *Sphingomonas*, recognized for its capability to degrade a wide range of environmental organic pollutants (42, 43), got significantly enriched in this region, especially in station S17. Many *Sphingomonas* species possess genes enabling the mineralization of polycyclic aromatic hydrocarbons, allowing them to utilize compounds like phenanthrene, anthracene, and fluoranthene as energy sources (43, 44). The high abundance of *Sphingomonas* in this area is therefore likely attributed to contamination from maritime transportation and oil spills. Given that microbial communities in marine ecosystems exhibit spatial heterogeneity, increasing sampling density could indeed enhance the resolution of biogeographic patterns. In this survey, although the total transect spans ~4,000 km, our 22 surface seawater samples were systematically collected along a latitudinal gradient (35°N to 75°N) covering distinct hydrographic regimes, including the Sea of Japan, the Bering Strait, and the Arctic Ocean. Furthermore, Mantel’s tests revealed strong correlations between community dissimilarity and environmental characteristics distance (Fig. 2b), suggesting that our sampling strategy effectively captured environmentally driven community variation. Our current study focuses on a single-year data set to establish foundational insights, and multiyear and seasonal data would be incorporated to explore temporal patterns, causal relationships, as well as to enhance the depth of analyses in our future research.

### Environmental factors in shaping microbial communities

Microorganisms play a critical role in ocean biogeochemical cycles, making it essential to investigate the factors driving variations in microbial community composition across different oceanic regions. Although sampling remote regions, such as those in the Arctic Ocean, poses significant challenges, large-scale studies are necessarily essential to identify microbial community differences and reveal underlying mechanisms (45, 46). In our extensive survey, which included 22 sampling stations spanning over 4,000 km across the Arctic Ocean, Chukchi Sea, Pacific Ocean, and Sea of Japan (35–76°N, 160°W–130°E), we aimed to explore key environmental factors shaping microbial communities.

In line with previous research on marine ecosystems, temperature emerged as the primary environmental factor influencing microbial community composition (47–49). Temperature’s impact on microbial communities is well-documented in other ecosystems, including soils, animal guts, and plant rhizospheres, where it is known to drive community structure through effects on metabolic rates and physiological thresholds (45, 50–53). In this study, taxa associated with warmer environments (e.g., the phyla Acidobacteria and Patescibacteria and the genus *Sphingomonas*) were significantly enriched in the Sea of Japan, while the richness index (Chao1) positively correlated with temperature, both of which contributed to observed shifts in microbial community structure.

Besides temperature, variations in seawater pH, potentially influenced by seasonal events such as ice melting, algal blooms, and anthropogenic activities (54, 55), also played a significant role in structuring prokaryotic communities in our samples. Together, these findings underscore the central importance of temperature and pH as environmental filters in microbial community assembly across oceanic regions.

### Deterministic and stochastic processes in community assembly

Understanding the ecological mechanisms that govern prokaryotic biogeography is essential for explaining variations in ocean microbiota composition and their implications for global ecosystem functions (56, 57). Our findings suggest that stochastic processes, including ecological drift, random dispersal, and historical contingencies, largely govern prokaryotic community assembly at broad spatial scales in marine environments. Microorganisms’ small size and high dispersal potential facilitate their widespread transport via ocean currents, wind, and animal vectors, thereby amplifying the influence of drift and dispersal on community structure (58, 59). Additionally, the high metabolic flexibility, broad physiological tolerance, rapid growth rates, and adaptability of microbes through horizontal gene transfer (60) buffer communities against strong local environmental filtering.

However, in specific regions like the Arctic Ocean, microbial communities exhibited higher phylogenetic similarity than expected (average βNTI < −2), indicating a strong role for deterministic homogeneous selection (19). This pattern likely results from the Arctic’s extreme environmental conditions, which impose selective pressures that favor the persistence of similar, adapted microbial taxa. Elevated NTI values in these communities further suggest deterministic selection by environmental filtering, where phylogenetically related taxa are more likely to thrive in the same environment due to similar adaptive traits (17). This effect is consistent with observations from the Barents Sea and Nansen Basin, where cold-adaptive microbes were selected in response to low-temperature environments (8). Such deterministic selection was also reported in the Barents Sea and Nansen Basin, where temperature acted as a strong environmental filter in selecting cold-adapted microorganisms. Temperature’s role as a primary environmental variable was further supported by our dbRDA analysis, showing its influence on microbial phylogenetic turnover across all samples (Fig. 5c). Together, these findings demonstrate the intricate balance of deterministic and stochastic processes in microbial community assembly, which should be analyzed at both regional and global scales.

### Conclusion

This study highlights the complex dynamics of marine prokaryotic communities from the Arctic Ocean to the Sea of Japan, shaped by distinct environmental factors and regional characteristics. Analysis of microbial diversity across 22 stations revealed a latitude-related gradient in alpha diversity, with a general decline in diversity with increasing latitudes, except in the Arctic regions. This deviation is likely due to the specific adaptations of Arctic microbial communities to extreme environmental conditions and broader temperature tolerance limits. The microbial communities in different regions further demonstrate microbes’ notable adaptations to local conditions in different marine regions. In particular, the cold-adapted oligotrophs such as *Planktomarina* and the SAR11 clade thrived in the Arctic Ocean, reflecting their adaptation to nutrient-poor, low-temperature environments. In contrast, microbes like *Sphingomonas*, known for degrading organic pollutants, were enriched in the Sea of Japan, where they likely respond to regional pollution stressors. The environmental variables, particularly temperature and salinity, were revealed to be the significant factors in explaining these differences in community compositions among samples. Besides, although the stochastic processes dominate the assembly of microbial phylogenetic diversity at broad spatial scale, temperature showed a primary influence on the phylogenetic turnover across all samples. This was especially evident in the Arctic Ocean, where deterministic homogeneous selection appeared to shape microbial communities, favoring specific taxa well-suited to Arctic conditions. Overall, these findings collectively highlight the central role of temperature in shaping marine microbial communities, influencing their diversity, composition, and assembly processes across marine environments.

## Data Availability

The sequence data presented in this study have been deposited in the NCBI database under accession number PRJNA1182108.
